# Advances in analyzing RNA diversity in eukaryotic transcriptomes: peering through the Omics lens

**DOI:** 10.12688/f1000research.9511.1

**Published:** 2016-11-14

**Authors:** Sushant Bangru, Auinash Kalsotra

**Affiliations:** 1 Department of Biochemistry, University of Illinois at Urbana-Champaign, Illinois, USA; 2 Institute of Genomic Biology, University of Illinois at Urbana-Champaign, Illinois, USA; 3 College of Medicine, University of Illinois at Urbana-Champaign, Illinois, USA

**Keywords:** RNA variants, Alternative splicing, next generation sequencing, mRNA modifications, computational pipelines, post-transcriptional gene regulation

## Abstract

Alternative splicing, polyadenylation, and chemical modifications of RNA generate astonishing complexity within eukaryotic transcriptomes. The last decade has brought numerous advances in sequencing technologies that allow biologists to investigate these phenomena with greater depth and accuracy while reducing time and cost. A commensurate development in biochemical techniques for the enrichment and analysis of different RNA variants has accompanied the advancement of global sequencing analysis platforms. Here, we present a detailed overview of the latest biochemical methods, along with bioinformatics pipelines that have aided in identifying different RNA variants. We also highlight the ongoing developments and challenges associated with RNA variant detection and quantification, including sample heterogeneity and isolation, as well as ‘Omics’ big data handling.

## How did we get here?

**Figure 1.**
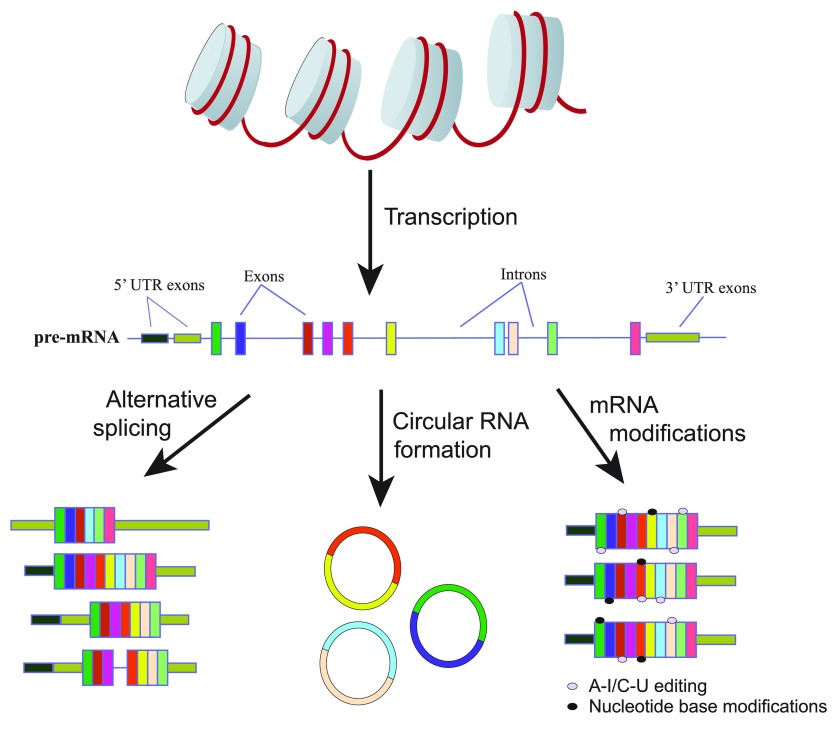
Alternative splicing, circular RNA formation, and mRNA modifications are the major mechanisms generating much of the mRNA diversity. Alternative splicing results in the selective inclusion or skipping of particular exons as well as alternate 5′ and 3′ untranslated region (UTR) selection within a processed mRNA transcript. In some cases, alternative splicing also results in intron retention events. Circular RNA formation occurs because of back splicing of the 3′ end of a downstream exon to the 5′ end of an upstream exon. Lastly, mRNAs can be modified through editing mechanisms converting A and C nucleotides to I and U nucleotides, respectively, as well as through the addition of functional groups on either the base or the sugar moiety of a nucleotide.

The RNA world hypothesis, a widely prevailing idea among molecular biologists, describes self-replicating RNA as the precursor to all modern life forms. Besides recognizing its primordial origin, we have come to realize that RNA is a highly complex and diverse macromolecule, which plays central roles in protein synthesis by serving as the encoder (mRNA), the decoder (tRNA), and the catalyst (rRNA). But our understanding of RNA diversity, form, and function continues to evolve as we keep discovering new and exciting classes of RNAs that carry out unexpected functions. Recent transcriptome studies, which capture nearly every transcript in a cell, have revealed an overwhelming number of RNA variants resulting from multiple transcription initiation sites, alternative pre-mRNA splicing and polyadenylation, post-transcriptional editing, and direct chemical modifications of RNA ( Figure 1). These studies have demonstrated that the variants play key roles in generating mRNA diversity by altering their coding sequences, half-lives, and translation efficiencies, allowing complex organisms to control temporal and tissue-specific transcriptome patterns.

**Figure 2.**
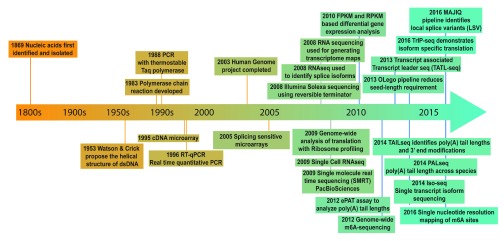
Timeline outlining the recent advances in analyzing RNA variants.

The systematic study of RNA variants began in the 1990s when cloning of expressed sequence tags revealed the diversity of mRNA populations expressed in different cells and tissues of metazoans ^1^. The advent of microarrays in the early 2000s expedited the discovery of new RNA variants ^2, 3^, but the technology had several limitations, including (i) high background levels due to dependence on hybridization, (ii) reliance on existing sequence knowledge, and (iii) a limited range of detection. In the last decade, the field has taken a significant leap forward with the development of next-generation sequencing. The timeline of major milestones that facilitated the discovery of novel RNA variants is provided in Figure 2.

First-generation sequencing required separate reactions for individual nucleotides and was limited by a gel electrophoresis step, which was further complicated by the need to use radioactive reagents ^4, 5^. Next-generation sequencing, however, uses advanced optics and flow cells to identify nucleotide sequences rapidly, making the analysis and sequence determination quicker and more accurate ^6– 8^. A number of next-generation sequencing platforms have been developed, and the most popular ones are Illumina Solexa ^9^ (relies on fluorescence imaging post-nucleotide addition every cycle, approximately 100 nucleotide reads, 0.1% error rate), Roche 454 sequencing ^10^ (long reads and short homopolymeric stretches sequenced in single-cycle, approximately 500 nucleotide reads, 1% error rate), Ion Torrent ^11^ (based on detection of H ^+^ ion release due to polymerization of dNTP probes, approximately 200 nucleotide reads, 1% error rate), and SOLiD sequencing ^12^ (relies on sequencing by ligation over synthesis, 35 nucleotide reads, less than 0.1% error rate). Of the above methods, the first three are classified as sequencing by synthesis methods whereas SOLiD is under the sequencing-by-ligation category. The field of sequencing by ligation has made rapid strides with the development of complete genomics technology that uses combinatorial synthesis methods, involving the use of multiple probe-anchor combinations to determine sequence ^13^. Additionally, Pacific Biosciences has recently introduced single-molecule real-time sequencing ^14^ of full-length transcripts, and the average read length is 2.5 kb (non-stop sequencing and real-time imaging, more than 1% error rates).

Perhaps the most significant breakthrough in the field was the development of massively parallel cDNA sequencing, commonly referred to as RNA-seq ^15^. It involves sequencing of cDNA fragments generated from total RNA after oligo-dT or random priming. For most transcriptome studies, mRNA is enriched from the total RNA pool by using either a poly(A) pull-down or selective depletion of rRNA. Following sequencing, the reads are aligned to a pre-annotated reference genome or assembled *de novo* to provide both the qualitative and the quantitative information of entire transcriptomes. This technique now can be used to identify new transcripts without having *a priori* knowledge of all gene models ^16^. Recent mapping tools can also identify splice junctions by using reads spanning across two exons, bolstering identification of alternative splice isoforms. Initially, RNA sequencing (RNA-seq) studies examined only the steady-state levels of RNA populations; however, the relative contribution of variable transcription (synthesis) and RNA degradation (stability) in those studies was unclear. New techniques such as Net-Seq ^17, 18^ and BRU-seq ^19^ address this problem. Net-Seq employs DNA-RNA-RNA polymerase complex stability to isolate 3′ ends of nascent transcripts. Bru-Seq uses pulse-chase labeling of newly transcribed RNA with bromouridine to determine rates of RNA synthesis and stability.

The past five years have witnessed a surge of new techniques employing RNA-seq to identify many new RNA variants, and isolation/enrichment methods are based on each variant type. In parallel, significant advances in computing power have expanded our ability to annotate and quantify these variants with high confidence from a single cell to complex tissues, along with the advent of consortium projects that have given rise to databases for their comparative and meta-analyses ^20– 22^. The increasing sensitivity of detection, wherein extremely minute quantities (picograms) of RNAs can be amplified and sequenced, has also spurred development of single-cell RNA-seq (scRNAseq) ^23^ for identifying heterogeneity within cell populations and uncovering transcriptome differences that may be hidden in bulk RNA-seq ^24^. Some of the commonly used sequencing technologies and bioinformatics packages for the detection of different RNA variants are provided in Table 1.

**Table 1.** Summary list of bioinformatics pipelines/tools for analyzing different RNA variants.

Variant analysis classification	Biochemical methods (methods for feature enrichment)	Bioinformatics tools
		Pre-processing tools	Analysis tools
Alternative splice variants	a. RNAseq post Poly(A) enrichment for mRNA pulldown b. Isoform sequencing (Iso-Seq) and SLR-RNA-seq ^95^ (synthetic long-read) for full-length transcript sequencing c. RNAseR enrichment for circular RNA	- Quality check: fastQC and HTSeq - Processing: Fastx, Trimmomatic, and cutadapt - Mapping: STAR ^96^, OLego ^97^, and TopHat ^98^	rMATS ^99^, MISO ^53^, IRCall, CIRCexplorer ^44^, MAJIQ ^37^, and DEXSeq
5′ and 3′ end variants	a. 3′ end enrichment libraries: 3P-Seq, PAPERCLIP, and 3′-Seq b. poly(A) tail length: TAIL-seq, PAL-seq, and LM-PAT c. 5′ end enrichment libraries: CAGE ^100^ and deepCAGE	- Quality check: fastQC and HTSeq - Processing: Fastx, Trimmomatic, and cutadapt - Mapping: STAR, OLego, and TopHat	MISO and DaPars
RNA modification variants	a. antibody-based IP: m6A, m1A, m5C, and hm5C b. Selective RNA chemistry: m5C and Ψ	- Processing: flexbar and Fastx - Mapping: STAR, OLego, and TopHat	MACS ^101^, HOMER ^102^, CIMS ^103^, and CITS ^104^
Translational variants	a. Ribosome profiling b. TATL-seq for alternative transcript leader sequences c. Poly(A)-primed sequencing (2P-seq) d. Frac-Seq and TrIP-seq	- Quality check: fastQC and HTSeq - Processing: Fastx, Trimmomatic, and cutadapt - Mapping: STAR, OLego, and TopHat	Plastid, RiboTaper ^105^, Cufflinks/Cuffdiff ^106^, and DESeq

In this review, we discuss different types of mRNA variants commonly found in metazoans, consider the latest advances in techniques to identify them, and provide a brief overview of the recent developments in bioinformatics pipelines for their quantitative analyses.

## Decoding the splice variants

Pre-mRNA splicing is an essential step for the expression of most genes in metazoans. Splice sites within a pre-mRNA facilitate the splicing and ligation of introns and exons, respectively, producing a mature mRNA transcript. Alternative splicing involves differential usage of splice sites to yield multiple mRNAs from a single gene. The functional outcomes are increased proteome diversity and the introduction of premature termination codons to degrade mRNAs by nonsense-mediated decay ^25^ and to generate variability in untranslated regions (UTRs) which modulate mRNA translation efficiency, stability, and localization ^26, 27^. Given the direct and widespread impact of alternative splicing on eukaryotic gene expression, cataloging the full repertoire of splice variants within individual cell types, developmental stages, and different human diseases has been a focus of many research laboratories.

The initial RNA-seq studies for detecting splicing patterns suffered from short read lengths, limiting the reliable genomic alignment of the alternative exon-exon junctions. Clever computational approaches, however, overcame these limitations through the addition of all potential splice junctions to the reference genome and accounting for them within the alignment mix ^28– 30^. The results transformed our view of the extent and complexity of eukaryotic transcriptomes, as more than 95% of human multi-exon genes were found to have alternatively spliced isoforms ^31^. Importantly, calculating the number of mapped reads against individual exons plus each splice junction provided a direct measure of splicing efficiency that allowed precise quantification of isoform ratios.

The development of strand-specific and paired-end sequencing led to improved mapping, the discovery of pervasive anti-sense transcription ^32, 33^, and many novel splice variants in vertebrate species ^34, 35^. Emerging technologies are geared toward sequencing the full-length transcripts (for example, Iso-seq ^36^) such that variably spliced regions from different areas of a single gene can be accurately connected to provide information about the entire mRNA isoforms (that is, from the 5′ end to the poly[A] tail). There has also been a strong push to improve the existing algorithms to capture the full complexity of splicing patterns within existing RNA-seq data. For instance, Vaquero-Garcia *et al*. have recently developed a new computational pipeline to accurately detect, visualize, and quantify complex local splice variants that are tissue-specific or change dynamically across different experimental conditions ^37^. ****

In addition to the discovery of variably spliced regions, RNA-seq has been an incredible asset for the investigation of splicing intermediates and novel splice products. For instance, it led to the identification of recursive splicing where long introns require specific exon contexts and are spliced out in phases rather than the usual two-step process ^38, 39^. Recursive splicing uses cryptic sites within the intron, known as recursive splice (RS) site/ratchet points, which are surprisingly more conserved than the regular 5′ and 3′ splice sites. However, the identification of these RS sites required extensive deep sequencing with over a billion paired-end reads, without poly(A) selection in an attempt to capture pre-mRNA and nascent RNA transcripts ^40, 41^. Likewise, RNA-seq has renewed interest in studying circular RNAs (circRNAs). The discovery of circRNAs, though known for over two decades, remained mostly serendipitous ^42, 43^. Typical RNA-seq experiments fail to conserve RNA circularity because fragmentation and the absence of a poly(A) tail or free 5′ or 3′ end cause de-enrichment of circRNAs during most library preparations. However, the lack of exposed 5′ or 3′ ends also stabilizes the circRNAs, making them resistant to exonuclease-mediated degradation. The recent development of biochemical methods to enrich for circRNAs (for example, RNAseR treatment to digest single-stranded RNA) along with new computational strategies (CIRCexplorer ^44^) to mine the existing Encyclopedia of DNA Elements (ENCODE) Ribozero RNA-seq data has aided their *de novo* detection in humans, mice, and *Caenorhabditis elegans*. The results have revealed that most circRNAs are non-coding splice variants containing multiple exons located in the middle of genes that are often expressed in a tissue- and developmental stage-specific manner and that these variants may act as sponges to antagonize microRNA activity ^45, 46^.

## Cracking the terminal variants

Whereas the gene body variants in mRNAs promote proteome diversity, variability at the terminal ends is known to serve more of a regulatory role in protein synthesis. This realization has led to a strong surge in the development of new methodologies to identify all the 5′ and 3′ end variants within eukaryotic transcriptomes. For instance, recent methods have revealed that alternative transcription start sites (TSSs) are prevalent in the human genome and that approximately 30 to 50% of human genes have more than one promoter ^47^. A substantial number of these alternative TSSs alter the 5′ UTRs, in turn modulating the translational output of mRNAs ^48^. 5′ UTRs are known to comprise many regulatory features—5′ cap structure, internal ribosome entry sites, G-quadruplexes, and so on—that can drastically affect the ability of ribosomes to assemble and initiate translation. Apart from forcing the gain or loss of such regulatory features, alternative TSSs generate RNA variants with different upstream open reading frames (uORFs). Ribosome footprinting studies have uncovered a significant fraction of such short translated uORFs within 5′ UTRs that potently repress the translation of downstream ORFs ^49– 51^.

Apart from providing proper signals for mRNA 3′ end formation and poly(A) site selection, the 3′ UTR is considered to be a regulatory hub for gene regulation. Proper 3′ end formation is essential for nuclear export and stability of mature mRNAs and their efficient translation ^52^. Analysis of existing RNA-seq data with new computational pipelines (MISO and DaPars) ^53, 54^ or sequencing of the libraries enriched for 3′ ends of mRNAs (3P-Seq, 3′-seq, 3′READS, and PAPERCLIP) have documented that over 50% of human mRNAs express variable 3′ UTRs—generated by alternative cleavage, splicing, and polyadenylation. Importantly, these 3′ UTR variants are differentially expressed during differentiation, development, and disease ^55– 58^, and they often influence the accessibility of microRNAs and RNA-binding proteins (RBPs) to the 3′ UTR *cis-*regulatory elements affecting mRNA stability, localization, and translation efficiency ^59^.

Poly(A) tail length is another important 3′ UTR feature that impacts the post-transcriptional fate of mRNAs. Although some biochemical methods (LMPAT ^60^ and ePAT ^61^) can accurately measure the poly(A) tail lengths of individual genes, precise measurements at a genome-wide scale are hampered by difficulties in sequencing homopolymeric sequences longer than 30 nucleotides. However, new advances in poly(A) tail length profiling methods (TAIL-seq and PAL-seq) use specialized 3′ adapters, RNAse T1 treatment (digests the mRNA body but not the poly[A] tail), and the spike in controls in library preparation. These biochemical improvements in conjunction with refined machine-learning algorithms to analyze the intensity of raw fluorescence signals allow the precise estimation of poly(A) tail lengths. These new methodologies have started to provide the first high-resolution, global views of poly(A) tail length variants and their relevance to mRNA stability and translational efficiency in cells and tissues from a variety of species, including yeast, *Drosophila*, zebrafish, *Xenopus*, mouse, and human ^62– 64^. ****

## Marking the variants

Though originally discovered in highly abundant rRNAs, tRNAs, and snRNAs, newly tailored mRNA sequencing methods have revealed that base modifications and editing sites are also highly prevalent in mRNAs ^65^. The study of mRNA modifications (methylation, hydroxy-methylation, and pseudouridylation) plus editing (A–I or C–U) is now commonly referred to as “epitranscriptomics” ^66– 68^. Much like the epigenome, the epitranscriptome expands the information content of nucleic acids, rendering them additional structural and functional flexibility. Recent advances have led to the profiling of millions of modification sites for each of the known mRNA modifications, including methylation, as well as base editing (such as A–I or C–U) ^69, 70^. Whereas most of these have been observed across all genes, some are limited to specific classes of transcripts.

Remarkably, the genome-wide mapping of modifications and editing sites has not only expanded the landscape of this new class of mRNA variants within eukaryotic transcriptomes but also unraveled their dynamics in abundance and site selection in response to intra- and extra-cellular stimuli ^71– 74^. The impact of these post-transcriptional marks is now recognized in almost every step of gene regulation, including mRNA processing, stability, and translation ^75^. An interesting example is the recent work on ADAR (adenosine deaminase acting on RNA) enzymes, showing their role in protecting double-stranded RNA (dsRNA) against recognition by dsRNA response pathways ^76^. Importantly, the writers, erasers, and reader proteins for some of these modifications have been identified, and the phenotypic and molecular consequences of their gain or loss of function have been demonstrated ^77^.

Today, the transcriptome-wide mapping of modified nucleosides relies on either antibody-based immunoprecipitation (m6A, m1A, m5C, and hm5C) ^66, 78^ or a modification-selective RNA chemistry approach (m5C and Ψ) ^79, 80^ to enrich for the target modification during library preparation. However, further optimization of these methods is needed to simultaneously obtain single-nucleotide resolution and stoichiometry of RNA modifications, as fractional modification represents another mechanism to generate functional diversity within individual mRNA pools. Current epitranscriptome profiling techniques accurately identify the modification sites within RNA transcripts but fail to provide information about the modified fraction for each site. Given that a modification may alter the mRNA secondary structure or binding site for RBPs, quantitative fractional information about each modified site in the transcriptome is highly desired.

## Translating the variants

Early dogma predicted that the amount of mRNA for a given gene directly correlated with the amount of protein made. However, we now know that through sequence variations discussed above and along with other RNA control elements, the translation of specific transcripts can be fine-tuned for the purpose of responding to cellular needs in a variety of contexts ^81^. Therefore, estimating average gene-level translation by ribosome profiling ^82^—a technique that uses short, approximately 28 nucleotide ribosome footprints on mRNA as a means of quantifying protein output—may not provide an accurate measure of the translational potential for individual RNA variants or isoforms. This is especially true for splice variants, which can exhibit variable coding capacities because of (i) sequence differences, (ii) altered mRNA half-lives, or (iii) differential nucleo-cytoplasmic export. Indeed, several recent efforts to measure isoform-specific translation have revealed discrete effects of 5′ end ^83^, 3′ end ^84^, and coding sequence ^85, 86^ diversity.

Using translation-associated transcript leader sequencing (TATL-seq), Arribere and Gilbert successfully mapped transcript leader boundaries across the yeast genome and discovered hundreds of new alternative transcript leader variants, the majority having differential translation efficiencies ^83^. Complementing this work, Spies *et al*. acquired decay and translation rates for proximal and distal 3′ UTR variants using poly(A)-primed sequencing (2P-seq) but found only a modest regulatory influence for alternative 3′ UTR sequences ^84^. By comparing isoform ratios in cytoplasmic and polyribosomal extracts from human cells (Frac-seq), Sterne-Weiler *et al*. observed that not all splice isoforms are similarly loaded into polyribosomes and that the presence of microRNA binding sites or premature termination codons can greatly affect the loading of individual isoforms into polyribosomes ^85^. Similarly, a major impact of mRNA isoforms on protein output was observed by Floor and Doudna, who recently adapted the classic approach of polysome profiling to measure isoform-specific translation (TrIP-seq) ^87^.

## Where are we headed?

Emerging technologies have given us a powerful toolbox to detect and quantify RNA variants from simple unicellular organisms to complex vertebrates. These new techniques allow for previously unimagined access to compare transcriptome patterns across species, tissues, developmental stages, and disease states. We now can isolate and sequence picogram amounts of RNA from fresh and formalin-fixed paraffin-embedded tissue specimens and even identify variants within single cells that are isolated from a wide range of biological samples. Moreover, the ENCODE project has generated unprecedented information about the many different RNA variants present in model organisms and human cells. Systematic discovery of *cis*-acting RNA elements, *trans*-acting RBPs that bind to these elements to control the production of variants, and their expression and subcellular localization is paving the way to uncovering the underlying code for generating much of the RNA diversity ^88, 89^. If sequencing costs continue to decline as anticipated, clinical measurements of normal and aberrant RNA variants in body fluids or tissue biopsies in the not-so-distant future may become part of personalized medicine, making for more accurate diagnoses and offering better-informed treatment plans.

However, as with most opportunities, certain challenges must be overcome to realize their full potential. What are the common challenges facing global profiling of RNA variants? First, biological specimens are usually heterogeneous, composed of multiple cell types. Since most RNA variants are expressed in a single cell type-specific ^90^, developmental stage-specific ^91^, or disease-specific ^92^ manner, particular attention must be paid when selecting optimal biological specimens for sequencing. For instance, individual cell types from most tissues now can be isolated by serial dilution, fluorescence-activated cell sorting, or laser-capture micro-dissection. Therefore, developing compound protocols that combine cell type-specific purification strategies with sequencing may provide more reliable information on RNA variants within particular cell types under normal or disease states. Second, identifying RNA variants from single-cell RNA-seq often requires multiple amplification steps to produce a sufficient quantity of nucleic acid for sequencing. These amplification steps introduce stochastic bias for certain sequences, which may cause dropout of certain low-abundance variants while amplifying others, thereby reducing the accuracy, reproducibility, and quantification power of detecting variants within single cells ^93^. Third, there has been a rapid surge in the deposition of transcriptome data in public repositories in recent years. This has resulted in new challenges about how to effectively integrate the RNA variant information with other “Omics” datasets and build streamlined platforms for metagene analyses. Finally, perhaps the biggest challenge facing the field is the storage, distribution, and analysis of massive amounts of sequencing data. Multiple consortium projects like ENCODE, TCGA (The Cancer Genome Atlas), ExAC (Exome Aggregation Consortium), and other individual centers generate sequencing data at an astounding pace, doubling their numbers every seven months ^94^. As the world moves toward a future of personalized genomic diagnostics and medicine, Stephens *et al*. propose that real-time transcript expression level analysis and the development of genomic variant databases against reference genomes will help solve the data storage problem ^94^. With growing data, there is a need for platform development for data distribution. Cloud computing-based analysis may serve as the most practical option. While sequencing-based variant analysis provides a big boost for the future of precision medicine, handling genomics data is one of the most pressing challenges for the next decade.
